# Carnitine and congeners as regulators of tumour necrosis factor

**DOI:** 10.1155/S0962935193000663

**Published:** 1993

**Authors:** Claudio De Simone, Edoardo Arrigoni Martelli, Piero Foresta


Mediators of Inflammation 2, S11–S16 (1993)

### Bacterial endotoxins: biological properties and mechanisms of action

C. Galanos[CA] and M. A. Freudenberg

Max-Planck-Institut für Immunbiologie 7800 Freiburg, Stübeweg 51, Germany

[CA] Corresponding Author

**ENDOTOXINS (lipopolysaccharides, LPS) are agents of pathogenicity of Gram-negative bacteria, implicated in the development of Gram-negative shock. Endotoxin reacts with lipopolysaccharide-sensitive cells producing endogenous mediators such as tumour necrosis factor alpha (TNFα). Macrophages are cells mediating the toxic activities of LPS and TNFα is the primary mediator of the lethal action of endotoxin. This review article discusses the various mechanisms by which endotoxin hypersensitivity in bacteria-sensitized animals develops. The paper concludes with a discussion on the possible protective effect of carnitine congeners against the lethal action of LPS.**

**Key words:** Bacterial endotoxins, Carnitine congeners, Lipopolysaccharide (LPS), Tumour necrosis factor alpha (TNFα)

### Introduction

Endotoxins are constituents of the outer membrane of Gram-negative bacteria. Isolated endotoxin administered into experimental animals elicits a large spectrum of biological activities which are also manifested during Gram-negative septic shock.

Endotoxins are lipopolysaccharides (LPS). In *Enterobacteriaceae* and in many cases of other Gram-negative bacteria, LPS are found to consist of three covalently linked regions, the lipid A, the core oligosaccharide and the O-specific polysaccharide. The structure and composition of the O-polysaccharide is highly variable among Gram-negative bacteria, determining the serological specificity of the parent bacterial strain. The core oligosaccharide is less variable in its structure and composition, a given core structure being common to large groups of bacteria. Lipid A is structurally the least variable part of the LPS molecule, exhibiting a similar structure and composition among many Gram-negative bacteria (for reviews see References 1–3). All three parts of the LPS molecule are immunogenic, eliciting the formation of antibodies interacting specifically with distinct epitopes in the respective region. The biological activity of LPS resides solely in the lipid A, the polysaccharide being devoid of toxic activity.[2] Table 1 summarizes the large spectrum of biological activities that were found to be expressed by purified LPS or isolated free lipid A. As seen from the table the activities of endotoxin are not always harmful but some of these, such as induction of tumour necrosis and adjuvant activity, can be beneficial to the host.

The biological activities of LPS are not direct effects of the LPS molecule but are induced indirectly by endogenous mediators that are produced following interaction of endotoxin with

**Table 1**. Biological activities of lipopolysaccharides and free lipid A

| | |
|---|---|
| Pyrogenicity | Induction of nonspecific resistance to infection |
| Lethal toxicity in mice | Induction of tolerance to endotoxin |
| Leucopenia | Induction of early refractory state to temperature change |
| Leucocytosis | Adjuvant activity |
| Local Shwartzman reaction | Mitogenic activity for cells |
| Bone marrow necrosis | Tumour necrotic activity |
| Embryonic bone resorption | Macrophage activation |
| Complement activation | Induction of colony stimulating factor |
| Depression of blood pressure | Induction of IgG synthesis in newborn mice |
| Platelet aggregation | Induction of protaglandin synthesis |
| Hageman factor activation | Induction of interferon production |
| Induction of plasminogen activator | Induction of tumour-necrotizing factor and other cytokines |
| *Limulus* lysate gelation | Induction of mouse liver pyruvate kinase |
| Toxicity enhanced by growing tumours | Type C RNA virus release from mouse spleen cells |
| Toxicity enhanced by BCG, *P. acnes* | Helper activity for Friend spleen focus-forming virus in mice |
| Toxicity enhanced by Gram-negative infection | Inhibition of phosphoenolpyruvate carboxykinase |
| Toxicity enhanced by adrenalectomy | Hypothermia in mice |
| Toxicity enhanced by D-galactosamine | |
| Enhanced dermal reactivity to noradrenaline | |

© 1993 Rapid Communications of Oxford Ltd

LPS-sensitive cells. Macrophages are cells mediating the toxic activities of LPS[4-6] and tumour necrosis factor alpha (TNFα) is a primary mediator of the lethal action of endotoxin.[7-10]

The activity of endotoxin may be influenced (enhanced or suppressed) by a number of plasma proteins of the host, which are capable of binding LPS. These include high and low density lipoproteins (HDL and LDL),[11-14] LPS-binding protein (LBP)[15] and, in addition, specific antibodies directed against the LPS serotype in question and which may be present in the individual host. In the case of endotoxin shock resulting from infection, the toxic activity of the LPS released from the infecting micro-organism may be influenced additionally by bacterial proteins (e.g. Omp A) with which the released LPS may be associated.[16]

Sensitivity to endotoxin is genetically determined, rabbits, swine and humans being highly sensitive; while mice, rats or guinea-pigs are, in comparison, much less sensitive. Mice, depending on strain, usually succumb to the lethal activity of 200–400 μg of LPS. Endotoxin-resistant strains of mice have been identified which are insensitive to all LPS effects.[17,18] These are usually referred to as LPS nonresponders and are designated as $lps^d$, in contrast to mice with normal sensitivity (LPS responder) which are designated as $lps^n$. The high resistance of $lps^d$ mice is due to a genetic defect in the LPS gene locus present on chromosome 4.[18]

### Endotoxin hypersensitivity

Although sensitivity to endotoxin is genetically determined it has been known for many years that the sensitivity of normal healthy animals may be considerably increased under different experimental conditions. The most important of these are listed in Table 2. Thus treatment of experimental animals with hepatotoxic agents such as D-galactosamine will increase their sensitivity to the lethal effects of endotoxin more than 100 000-fold. A significant degree of sensitization may also be achieved following treatment of mice with muramyl dipeptide (MDP), a partial structure of peptidoglycan. Further, adrenalectomy hypophysectomy or exposure to a hyperthermic environment will all enhance considerably the sensitivity to endotoxin.[19]

The sensitivity of mice to endotoxin was also found to be increased by a number of growing tumours. Thus Lewis lung carcinoma, and the EMT6 sarcoma growing in C57BL/6 and BALB/C mice, respectively, were shown to increase considerably the endotoxin sensitivity of the animals[20] (Table 3).

Sensitization to endotoxin also proceeds following treatment with live (infection) or killed bacteria.[21,22] Both Gram-positive[23] and Gram-negative micro-organisms were shown to increase the susceptibility of mice to the lethal activity of endotoxin. Sensitization by bacteria is of special interest and is dealt with in more detail in the following section.

**Table 2.** Induction of hypersensitivity to endotoxin treatment

| Condition which increases endotoxin hypersensitivity | Sensitization factor |
|---|---|
| Microbial infections | |
| Gram-negative | |
| *Salmonella* | |
| *E. Coli* | |
| *Klebsiella* | |
| *Coxiella burnetii* | 100–>1 000 |
| Gram-positive | |
| *Propionibacterium acnes* | |
| BCG | |
| Bacterial products | |
| Proteins | 50 |
| MDP | 100 |
| Parasitic infections | |
| Malaria (*B. chabaudi chabaudi*) | >100 |
| Growing tumours | |
| Lewis lung carcinoma | >10 000 |
| EMT6 sarcoma | 200 |
| Hepatotoxic agents | |
| D-galactosamine | 100 000 |
| α-amanitine | >1 000 |
| Other agents | |
| Carbon tetrachloride | >1 000 |
| Lead acetate | >1 000 |
| Actinomycin-D | >10 000 |
| Hyperthermia | |
| Environmental temperature 30–33°C | >1 000 |
| Cortisone deficiency | |
| Adrenalectomy | >1 000 |
| Hypophysectomy | >1 000 |

**Table 3.** Hypersensitivity of LLC and EMT6 tumour bearing mice to LPS or recombinant TNF

| Mice | $LD_{50}$(μg/mouse) LPS | LPS |
|---|---|---|
| Normal C57BL6 | 400 | 300 |
| C57BL with carcinoma (15 day tumour) | 0.1 | 0.01 |
| Normal Balb/c | 300 | 300 |
| Balb/c with sarcoma (15 day tumour) | 2 | 4 |

Female C57BL/6 and Balb/c mice were inoculated respectively with 3LL cells intramuscularly or EMT6 cells subcutaneously. Fifteen days later the animals were challenged intraperitoneally with increasing doses of recombinant TNF or *Salmonella a. equi* LPS. Each group was formed of six animals receiving increasing doses of the test compound. The mortality was recorded during 48 h.

### Bacteria-induced hypersensitivity to endotoxin

For the induction of hypersensitivity to LPS by bacteria both live or killed micro-organisms may be

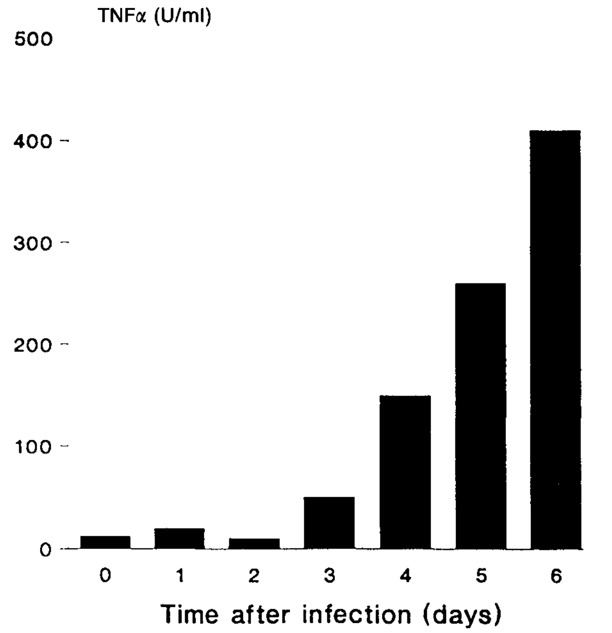

FIG. 1. Enhanced production of TNFα by LPS in C3H/HeN mice infected with *S. Typhimurium*. Mice were infected with 50 CFU *S. typhimurium* and challenged with 10 μg LPS. Plasma was collected 60 min after LPS administration.

used. Considerable changes in sensitivity of C57BL/6 mice to the lethal activity of LPS following infection with a lethal inoculum of *Salmonella typhimurium* have been demonstrated.[24] Thus, before infection, a dose of over 200 μg endotoxin was required to kill the animals. After infection, sensitivity increased daily in an almost logarithmic pattern and by day 5 after infection the lethal dose of endotoxin was less than 1 μg.[24]

Sensitization to LPS may be also achieved by sub-lethal infection as shown in Fig. 1.[22] Mice (C3H/Tif), infected with a sublethal inoculum of *S. thyphimurium* exhibit enhanced sensitivity to endotoxin. Sensitization becomes evident on day 2 after infection, reaches a maximum on days 7 to 8 and decreases again reaching normal levels several weeks later. Fig. 1 also shows that sensitization to LPS by sublethal infection is at the same time a sensitization to TNFα.

### Mechanisms of endotoxin hypersensitivity induced by bacteria

The property of bacteria to enhance endotoxin sensitivity is not confined to *S. typhimurium*, but is a general phenomenon observed with different live or killed Gram-negative and Gram-positive bacteria. An example of this is shown in Table 4.

Mice made hypersensitive to the lethal effects of LPS by bacteria are found, on LPS challenge, to produce considerably more TNFα than do normal animals.[25] This is shown in Table 5 where it can be seen that treatment of mice with *Propionibacterium acnes* or *S. typhimurium* leads, on LPS challenge, to

**Table 4.** Sensitivity of mice pretreated with different bacteria to the lethal effects of LPS

| Pre-treatment | Approx. $LD_{50}$(μg LPS) Mice HeN | Sn |
|---|---|---|
| none | 400 | 100 |
| *P. acnes* | 0.2 | 0.1 |
| *C. burnetii* | 2 | 0.5 |
| *S. typhimurium* | 3 | 3 |

**Table 5.** Enhanced production of TNFα by LPS in C57BL/10 ScSn mice treated with bacteria

| Treatment | | (ng/ml) |
|---|---|---|
| none | none | n.d. |
| | 10 | 2.1 |
| *P. acnes* | none | n.d. |
| | 10 | 3 000 |
| *S. typhimurium* | none | n.d. |
| | 10 | 454 |

Mice were treated with *P. acnes* (500 μg, 7 days before challenge) I.V. or *S. typhimurium* (50 CFU, 3 days before challenge) i.p., and challenged with LPS i.v. Serum for TNFα assay was collected 1 h after challenge. TNFα was measured by the 929 cell cytotoxicity assay with murine rTNFα as standard. n.d. = not detectable a 1 500- and 200-fold increase in the amount of TNFα produced, respectively (Fig. 2).

Since TNFα is a primary mediator of the lethal activity of LPS, the overproduction of TNFα by

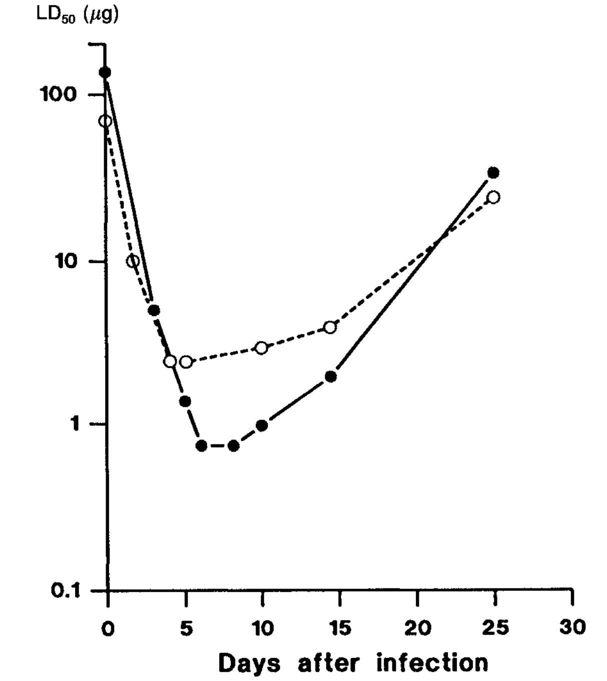

FIG. 2. Sensitization of mice to the lethal activity of LPS and HrTNFα by sub-lethal infection with *S. typhimurium*. Mice (C3H/Tif) were infected with 2 × $10^4$ CFU of *S. typhimurium* and on different days thereafter groups were challenged with different amounts of LPS and the $LD_{50}$ values were calculated. ● LPS, ○ HrTNFα.

Table 6. Effect of treatment with bacteria on the sensitivity of mice to the lethal activity of human rTNFα

| Treatment | | Lethality % |
|---|---|---|
| none | 75 | 0 |
| | 225 | 50 |
| *P. acnes* | 1 | 0 |
| | 5 | 20 |
| | 10 | 100 |
| *S. typhimurium* | 1 | 10 |
| | 10 | 100 |

C57BL/10 ScSn mice were treated with heat killed *P. acnes* (500 μg i.v., 7 days before challenge), or infected with *S. typhimurium* (50 CFU i.p., 3 days before challenge). Human TNFα was administered i.v.

LPS, in bacteria sensitized animals, would alone explain hypersensitivity. However, the high endotoxin sensitivity of bacteria treated mice is not due only to an overproduction of TNFα. Mice sensitized to the lethal effects of LPS by bacteria are also found to be hypersensitive to the lethal activity of TNFα. This is shown in Table 6. Therefore the hypersensitivity to the lethal effects of endotoxin seen in bacteria sensitized mice is based on (a) an overproduction of TNFα, and (b) a higher sensitivity to the lethal effects of TNFα.

The induction of hypersensitivity by Gram-negative bacteria is of special interest since these micro-organisms also produce endotoxin. The present results make it evident that Gram-negative bacteria not only produce endotoxin but also sensitize the infected organism to its toxic action, and therefore enable a better understanding of the hazardous consequences of Gram-negative infections.

### Mechanism of the sensitization to endotoxin by bacteria

*Interferon gamma, a mediator of the bacteria-induced sensitization:* Very recently a breakthrough in the understanding of the mechanism by which bacteria sensitize the organism to endotoxin was achieved. When mice are infected with live, or treated with killed, Gram-negative or Gram-positive bacteria, they are found to contain in their serum significant amounts of interferon gamma (IFNγ).[25] The production of IFNγ following treatment with bacteria is true for all strains of mice that are sensitive to endotoxin. The observation was made however that a similar treatment of LPS resistant ($lps^d$) strains of mice (see introduction) with bacteria does not lead to IFNγ production.[26] This observation suggested that the inability of $lps^d$ mice to be sensitized by bacteria might be due to their inability to produce IFNγ. This possibility was investigated closely, and evidence could be obtained that IFNγ is the mediator of sensitization of animals treated with bacteria to the lethal activity of LPS.[25] Thus, administration of anti-IFNγ monoclonal antibodies to mice pre-treated with bacteria inhibited the overproduction of TNFα (Fig. 3) and abolished the development of sensitization to the lethal activity of LPS (Table 7).

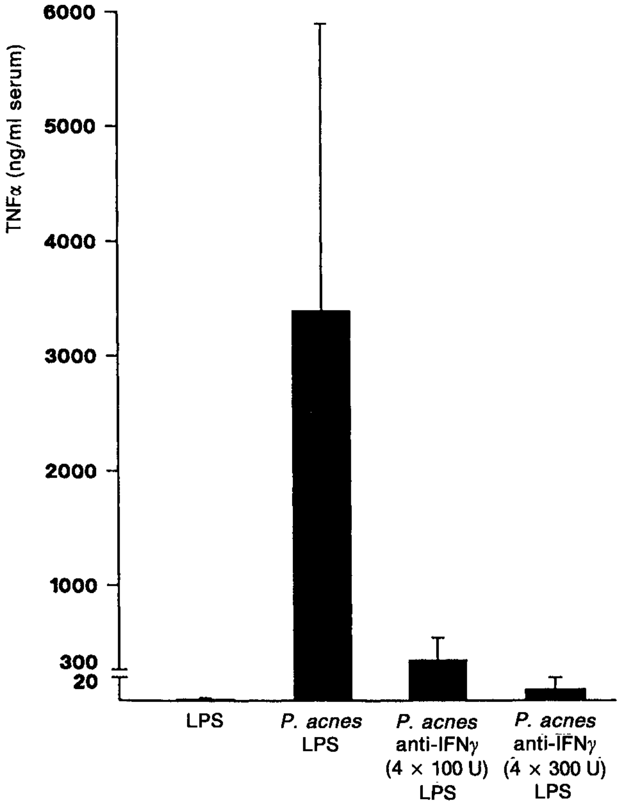

FIG. 3. Effect of anti-IFNγ on *P. acnes* induced sensitization to LPS. TNFα production.

Table 7. Effect of IFNγ antibodies on the *P. acnes* induced sensitization to LPS lethality

| | Lethality (dead/total) | | |
|---|---|---|---|
| LPS (μg) | Controls | *P. acnes* + IgG | *P. acnes* + mAb |
| 100 | 5/5 | — | — |
| 75 | 1/5 | — | — |
| 50 | 0/10 | — | 6/10 |
| 25 | — | — | 0/5 |
| 1 | — | 5/5 | — |
| 0.1 | — | 2/5 | — |
| 0.01 | — | 0/5 | — |

C57BL/10 ScSN mice received 500 μg *P. acnes*, i.v. Thereafter, one group of mice received 4 x 300 μg (300 U) anti-IFNγ, a second group 4 x 300 μg control IgG i.p. on days 0, 2, 4 and 6 after *P. acnes* treatment. Seven days after *P. acnes*, all mice were challenged with LPS, i.v. Normal, untreated Sn mice, injected with LPS only served as controls. Lethality was scored up to 72 h after LPS injection.

### Protection against endotoxin shock

Even since LPS was recognized as the main toxic component of Gram-negative bacteria, research groups all over the world have been searching for effective ways for treating and preventing endotoxin shock. One approach investigated extensively has been the use of antibodies to different regions of the LPS molecule. Of special interest have been antibodies directed towards parts of the LPS molecule that are common among clinically relevant Gram-negative micro-organisms. Other approaches include the use of cortisone, antibodies to the LPS receptor and the use of LPS receptor antagonists.

Recently, the authors investigated whether carnitine congeners might exhibit a protective effect against the lethal action of LPS. In these experiments both L-carnitine and acetyl-L-carnitine were used. The lethality models used include mice sensitized to the lethal activity of LPS by D-GalN and by *Propionibacterium acnes*, as well as normal mice. In approximately 50% of the experiments a protection was seen in both sensitization models. Even where no protection was found in terms of survival, a prolongation of survival was always evident. The following tables show the results of typical experiments in which protection was found.

Table 8 shows that administration of 5 mg acetyl-L-carnitine/mouse, 30 min prior to a lethal challenge with LPS and D-GalN afforded complete protection to the animals. A similar protection was seen when instead of LPS, recombinant hTNFα and D-GalN were used for challenge (Table 9). An investigation of the time of acetyl-L-carnitine administration that yields optimal protection revealed that the drug afforded maximum protection when administered 1–2 h before LPS/GalN challenge (Table 10). A protection by acetyl-L-carnitine was also seen in mice sensitized by *P. acnes* and challenged with LPS (Table 11). More experiments are being carried out in order to confirm the protection seen so far and to make a preliminary identification of the possible mechanisms involved.

**Table 8.** Effect of acetyl-L-carnitine on the lethal activity of LPS in D-GalN sensitized mice

| Ac-L-carnitine (mg) | D-GalN (mg) | LPS (μg) | Lethality dead/total |
|---|---|---|---|
| 5 | 20 | 0.002 | 0/4 |
| – | 20 | 0.002 | 4/4 |

Mice were C57BL/6 strain; LPS was from *S. abortus equi*; all injections were administered i.v.; acetyl-L-carnitine was administered 30 min prior to LPS/D-GalN

**Table 9.** Effect of acetyl-L-carnitine on the lethal activity of TNFα in D-GalN sensitized mice

| Ac-L-carnitine (mg) | D-GalN (mg) | TNF (μg) | Lethality dead/total |
|---|---|---|---|
| 5 | 20 | 1 | 0/4 |
| – | 20 | 1 | 4/4 |

Mice were C57BL/6 strain; LPS was from *S. abortus equi*; all injections were administered i.v.; acetyl-L-carnitine was administered 30 min prior to LPS/D-GalN

**Table 10.** Effect of time of acetyl-L-carnitine injection on the lethal activity of LPS/GalN in mice

| Time of Ac-L-carnitine administration (h) | Time of LPS/GalN injection (h) | Lethality deal/total |
|---|---|---|
| −24 | 0 | 1/4 |
| −4 | 0 | 2/4 |
| −2 | 0 | 0/4 |
| 0 | 0 | 1/4 |
| 1 | 0 | 2/4 |
| 4 | 0 | 4/4 |

C57BL/6 mice received acetyl-L-carnitine (5 mg) at the times indicated, and D-GalN (20 mg) with LPS (0.002 μg) at 0 h.

**Table 11.** Effect of acetyl-L-carnitine on the lethal activity of LPS in *P. acnes* sensitized mice

| Ac-L-carnitine (mg) | LPS (mg) | Lethality dead/total |
|---|---|---|
| 10 | 0.2 | 1/4 |
| 5 | 0.2 | 0/4 |
| 2.5 | 0.2 | 0/4 |
| 1.25 | 0.2 | 3/4 |
| – | 0.2 | 4/4 |

(C57BL/6) mice were treated with 500 μg killed *Propionibacterium acnes* i.v. 7 days before challenge. Acetyl-L-carnitine was administered i.p. 1 h before LPS (i.v.).

### References

1. Lüderitz O, Galanos C, Lehman V, Mayer H, Rietschel E Th, Weckesser J. Chemical structure and biological activities of lipid A's from various bacterial families. *Naturwissenschaften* 1978; **65**: 579–585.
2. Galanos C, Lüderitz O, Rietschel E Th, Westphal O. Newer aspects of the chemistry and biology of bacterial lipopolysaccharides, with special reference to their lipid A component. In: Goodwin TW, ed. *International Review of Biochemistry, Vol. 14, Biochemistry of Lipids II*, Baltimore: University Park Press, 239–335.
3. Rietschel E Th, ed. *Handbook of Endotoxin, Vol. 1, Chemistry of Endotoxin.* Elsevier, 1984.
4. Michalek SM, Moore RN, McGhee JR, Rosenstreich DL, Mergenhagen SE. The primary role of lymphoreticular cells in the mediation of host responses to bacterial endotoxin. *J Infect Dis* 1980; **141**: 55–63.
5. Rosenstreich DL, Vogel SN. Central role of macrophages in the host response to endotoxin. In: Schlesinger D, ed. *Microbiology—1980.* Washington, D.C. American Society for Microbiology, 1980; 11–15.
6. Freudenberg MA, Keppler D, Galanos C. Requirement for lipopolysaccharide-responsive macrophages in galactosamine-induced sensitization to endotoxin. *Infect Immun* 1986; **51**: 891–895.
7. Beutler B, Milsark JW, Cerami AC. Passive immunization against cachectin/tumor necrosis factor protects mice from lethal effect of endotoxin. *Science* 1985; **229**: 869–871.
8. Lehmann V, Freudenberg MA, Galanos C. Lethal toxicity of lipopolysaccharide and tumour necrosis factor in normal and D-galactosamine-treated mice. *J Exp Med* 1987; **165**: 657–663.
9. Galanos C, Freudenberg MA, Coumbos A, Matsuura M, Lehmann V, Bartoleyns J. Induction of lethality and tolerance by endotoxin are mediated by macrophages through tumour necrosis factor. In: Bonavida B, Gifford GE, Kirchner H, Old LJ, eds. *Tumour Necrosis Factor/Cachectin and Related Cytokines.* Basel: S. Karger, 1988: 114–127.

10. Freudenberg MA, Galanos C. Tumour necrosis factor alpha mediates lethal activity of killed gram-negative and gram-positive bacteria in D-galactosamine-treated mice. *Infect Immun* 1991; **59**: 2110–2115.
11. Skarnes RC. In: Berry LJ, ed. *Cellular Biology of Endotoxin, Vol. 3.* Amsterdam, New York, Oxford: Elsevier, 1985; 56.
12. Ulevitch RJ, Johnston AR, Weinstein DB. New function for high density lipoproteins. Their participation in intravascular reactions of bacterial lipopolysaccharides. *J Clin Invest* 1979; **64**: 1516–1524.
13. Freudenberg MA, Bøg-Hansen TC, Back U, Jirillo E, Galanos C. Interaction of lipopolysaccharides with plasma high density lipoprotein in rats. In: Eaker D, Wadstrom T, eds. *Natural toxins.* New York: Pergamon Press, 1980.
14. Munford RS, Hall CL, Lipton JM, Dietschy JM. Biological activity, lipoprotein-binding behaviour, and *in vivo* disposition of extracted and active forms of *Salmonella typhimurium* lipopolysaccharides. *J Clin Invest* 1982; **70**: 877–888.
15. Schuhmann RR, Leong SR, Flaggs GW, Gray PW, Wright SD, Mathison JC. Structure and function of lipopolysaccharide-binding protein. *Science* 1990; **249**: 1429–1431.
16. Freudenberg MA, Meier-Dieter U, Staehelin T, Galanos C. Analysis of LPS released from *Salmonella abortus equi* in human serum. *Microb Pathogen* 1991; **10**: 93–104.
17. Sultzer BM, Goodman GW. Characteristics of endotoxin-resistant low-responder mice. In: Schlesinger D, ed. *Microbiology—1977,* Washington DC: American Society for Microbiology, 1977: 304–309.
18. Coutinho A, Meo T. Genetic basis for unresponsiveness to lipopolysaccharide in C57BL/10Cr mice. *Immunogenetics* 1978; **7**: 17–24.
19. Galanos C, Freudenberg MA, Katschinski T, Salomão R, Mossmann H, Kumazawa Y. Tumour necrosis factor and host response to endotoxin. In: Ryan J, Morrison DC, eds. *Bacterial endotoxin lipopolysaccharides, Vol. 2. Immunopharmacology and pathophysiology.* Boca-Raton, FL: CRC Press Inc., 1992; 75–104.
20. Bartoleyns J, Freudenberg MA, Galanos C. Growing tumours induce hypersensitivity to endotoxin and tumor necrosis factor. *Infect Immun* 1987; **55**: 2230–2233.
21. Galanos C, Freudenberg MA, Matsuura M. Mechanisms of the lethal action of endotoxin hypersensitivity. In: Friedman H, Klein TW, Nakano M, Nowotny A, eds. *Endotoxin: Advances in experimental medicine and biology, Vol. 256.* New York: Plenum Press, 1990; 603–619.
22. Matsuura M, Galanos C. Induction of hypersensitivity to endotoxin and tumour necrosis factor by sublethal infection with *Salmonella typhimurium. Infect Immun* 1990; **58**: 935–937.
23. Suter E, Ullman GE, Hoffmann RG. Sensitivity of mice to endotoxin after vaccination with BCG (bacillus Calmette-Guérin). *Proc Soc Exp Biol Med* 1958; **99**: 167–169.
24. Galanos C, Freudenberg MA, Krajewska D, Takada H, Georgiev G, Bartoleyns J. Hypersensitivity to endotoxin. *EOS J Immunol Immunopharmacol* 1986; **6(Suppl. 3)**: 78–81.
25. Katschinski T, Galanos C, Coumbos A, Freudenberg MA. Gamma interferon mediates *Propionibacterium acnes*-induced hypersensitivity to lipopolysaccharide in mice. *Infect Immun* 1992; **60**: 1994–2001.
26. Freudenberg MA, Kumazawa Y, Meding S, Langhorne J, Galanos C. Gamma interferon production in endotoxin-responder and -nonresponder mice during infection. *Infect Immun* 1991; **59**: 3484–3491.
